# Diminished Expression of Galectin-3 Around Blisters in Bullous Pemphigoid: An Immunohistochemistry Study

**DOI:** 10.5826/dpc.1004a106

**Published:** 2020-10-26

**Authors:** Maryam Aghighi, Bruce R. Smoller

**Affiliations:** 1 Department of Pathology, Robert Wood Johnson Barnabas Health, Livingston, NJ, USA; 2 Department of Pathology, University of Rochester School of Medicine and Dentistry, Rochester, NY, USA

**Keywords:** bullous pemphigoid, galectin-3, blister formation

## Abstract

**Background:**

Bullous pemphigoid (BP) is a subepidermal blistering disorder caused by autoantibodies directed against hemidesmosomal proteins. Many patients with BP demonstrate circulating IgE autoantibodies. Although the role of IgE in the pathogenesis of BP is unknown, a correlation between IgE antibodies and eosinophilia has been observed. Soluble CD23 and galectin-3 are the main elements of the IgE group. The roles for CD23 in BP as a potential biomarker and IgE production regulator have been characterized, but no studies have evaluated any roles for galectin-3 in this disease.

**Objective:**

In this study, we evaluated galectin-3 expression in BP as a first step in assessing its role in the pathogenesis of this autoimmune blistering process.

**Patients and Methods:**

Sixty specimens diagnosed as BP were stained with antibodies to galectin-3. The percentages of nuclear and cytoplasmic galectin-3 expression and staining intensity were evaluated.

**Results:**

There was a significant difference in galectin-3 cytoplasmic and nuclear expression within keratinocytes immediately surrounding and above the blisters: (1) cytoplasmic (mean = 17.2% ± 2.4%) vs adjacent unaffected skin (mean = 66.7% ± 2.0%, P < 0.0001) and (2) nuclear (mean = 1.9% ± 0.4%) vs adjacent unaffected skin (mean = 13.2% ± 1.2%, P < 0.0001).

**Conclusions:**

Lower expression of galectin-3 around blisters in BP may suggest a role as an adhesion molecule. Loss of galectin-3 may add to the extension of blister formation by initiating cell-extracellular matrix disassembly and may be involved with the associated dermal inflammation and the eosinophil chemotaxis. Further studies will be necessary to elucidate the result of this observed loss on disease pathogenesis.

## Introduction

Bullous pemphigoid (BP) is a subepidermal disorder described by autoantibodies against hemidesmosomes in the dermal and epidermal junction, usually in elderly patients. BP usually starts with pruritis and urticaria and subsequently progresses to the development of blisters on the skin caused by subepidermal separation through the basement membrane at the level of the lamina lucida [1]. In addition to the IgG deposition seen along the dermal-epidermal junction (variably with depositions of IgM, IgA and C3, as well), most patients with BP present an autoimmune response by generating IgE autoantibody. Although the role of IgE in the pathogenesis of BP is unknown, a correlation between IgE antibodies and eosinophilia has been observed [2]. Soluble CD23 and galectin-3 are 2 main elements of the IgE group. While the role of soluble CD23 in BP as a potential biomarker, IgE production regulator and disease severity predictor has been characterized [3], no studies about putative roles for galectin-3 in BP have been performed [4]. Galectin-3 is a beta galactoside binding protein that is essential in the cell-to-cell or matrix adhesion. It broadly exists in the nucleus and cytoplasm of various cell types or may be found extracellularly on the cell surface [5]. As no studies have been reported on the role of galectin-3 immunohistochemical profile in pemphigoid disease, we attempted to evaluate the galectin-3 expression in patients with BP in the current study.

## Materials and Methods

This retrospective study was approved by the institutional review board of our institution. We included 60 specimens from 55 patients diagnosed with BP between 2017 and 2020. Five patients had 2 such biopsies.

The biopsy specimens were processed in formalin, dehydrated through graded alcohol washes, embedded in paraffin and sliced on a microtome. Tissue slices on glass slides were stained with Cell Marque mouse monoclonal galectin-3 (MilliporeSigma, Inc.) using an Autostainer Link 48 (Agilent Technologies, Inc) with Low pH antigen retrieval and EnVision FLEX detection kit. The antibody was used in a dilution of 1:25. The immunohistochemical analysis was performed under a light microscope (Olympus BX40F).

The percentage of galectin-3 nuclear and cytoplasmic expression around blister and adjacent unaffected skin were evaluated both qualitatively and semi-quantitatively by the authors and compared using a t-test. The intensity of galectin-3 around the blister and adjacent unaffected skin was evaluated semi-quantitatively using the range of 1+ to 4+ and compared using a t-test. The value of 1+ showed the lowest intensity with very light brown-colored stain, and 4+ showed the highest intensity with very dark brown-colored stain. Interobserver concordance was high, as staining differences were prominent. All statistical calculations were performed using Microsoft Excel software. An alpha level of P < 0.05 was used to indicate significant differences.

## Results

All included specimens had been previously diagnosed as BP based upon the patients’ clinical findings, histologic findings of a subepidermal blister that contained eosinophils in the blister cavity, and the demonstration of linear staining with IgG and C3 along the dermal-epidermal junction on contemporaneously received biopsies analyzed with direct immunofluorescence.

Galectin-3 showed lower expression above and lateral to the blisters compared to adjacent unaffected skin. We observed these changes in galectin-3 expression in both cytoplasmic and nuclear patterns around the blisters and adjacent unaffected skin. The galectin-3 expression was diffuse in adjacent unaffected skin with high intensity in both the cytoplasm and nucleus of keratinocytes. However, expression was diminished and irregular, with lower intensity above and lateral to the blisters. We observed a strong expression of galectin-3 in the inflammatory infiltrate and the surrounding vessels underlying the blisters, as well as the sebaceous glands near the blisters.

Out of 60 biopsies analyzed, this difference was noted in galectin-3 cytoplasmic expression of 56 biopsies (93.3%) and failed to demonstrate the difference in only 4 biopsies (6.7%). Similarly, galectin-3 nuclear expression of 59 biopsies (98.3%) demonstrated this difference, while only 1 biopsy (1.7%) did not show the difference.

**Figure 1:**
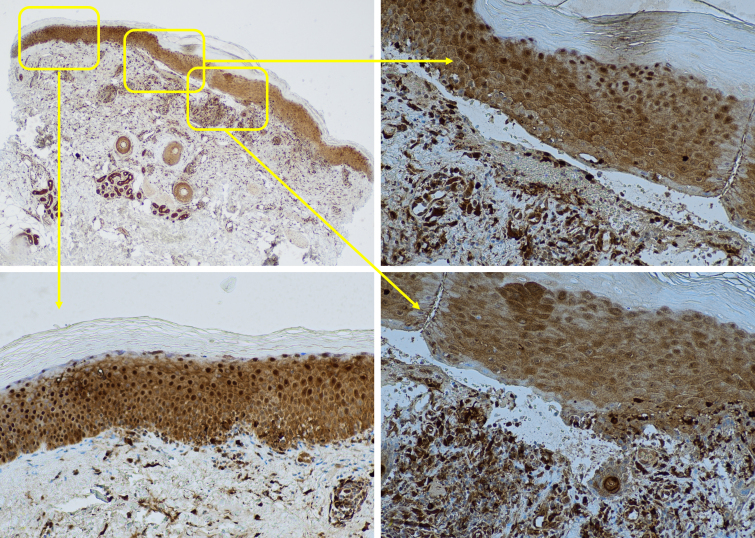
Lower expression of galectin-3 around the blister compared to adjacent unaffected skin. (A) Galectin-3 stain of a subepidermal blister of bullous pemphigoid (×20), (B) lower expression of galectin-3 above the blister (×200), (C) adjacent unaffected skin (×200), (D) lower expression of galectin-3 lateral to the blister (×200).

Figure 1, A–D illustrates an example in which 10% galectin-3 positivity is noted in a cytoplasmic pattern with the intensity of 1+ and 1% nuclear expression with an intensity of 1+ above (Figure 1 B) and lateral (Figure 1 D) to the blister. These were significantly lower than adjacent unaffected skin (Figure 1C), demonstrating 70% galectin-3 positivity in a cytoplasmic pattern with an intensity of 2+ and 10% nuclear expression with the intensity of 2+.

**Figure 2:**
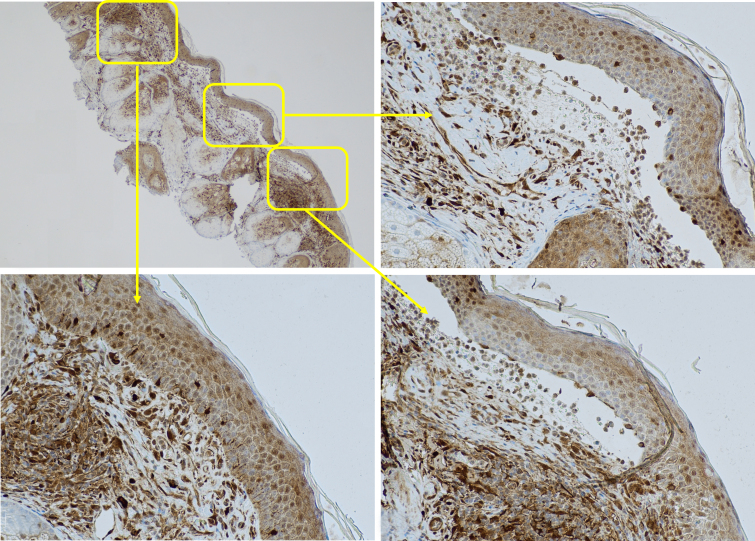
Lower expression of galectin-3 around the blister compared to adjacent unaffected skin. (A) Galectin-3 stain of a subepidermal blister of bullous pemphigoid (×20), (B) lower expression of galectin-3 above the blister (×200), (C) adjacent unaffected skin (×200), (D) lower expression of galectin-3 lateral to the blister (×200).

Figure 2, A–D demonstrates that galectin-3 was expressed in 5% and 1% in cytoplasmic and nuclear patterns respectively with the intensity of 1+ above (Figure 2B) and lateral (Figure 2D) to the blister, which were significantly lower than adjacent unaffected skin (Figure 2C) with 70% and 20% galectin-3 positivity in cytoplasmic and nuclear patterns respectively with the intensity of 3.

**Figure 3:**
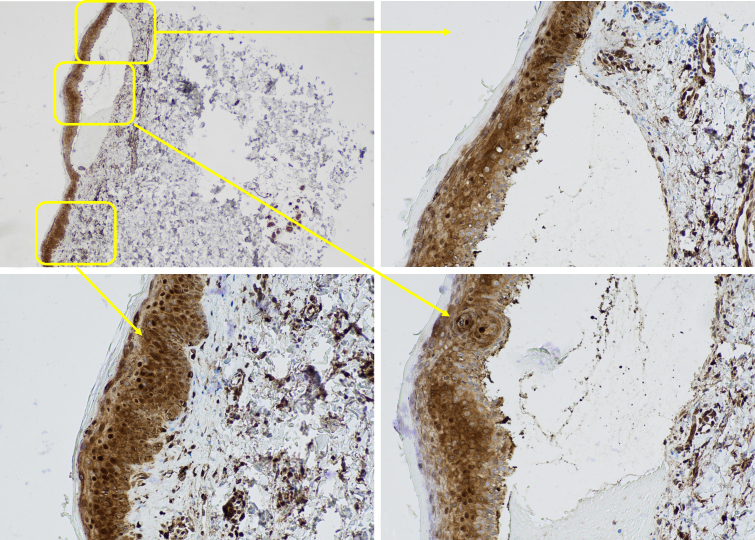
Lower expression of galectin-3 around the blister compared to adjacent unaffected skin. (A) Galectin-3 stain of a subepidermal blister of bullous pemphigoid (×20), (B) lower expression of galectin-3 lateral to the blister (×200), (C) adjacent unaffected skin (×200), (D) lower expression of galectin-3 above the blister (×200).

Similarly, Figure 3, A–D shows 10% cytoplasmic and 1% nuclear galectin-3 positivity with the intensity of 1+ above (Figure 3B) and lateral (Figure 3D) to the blister in cytoplasmic and nuclear patterns, which were significantly lower than adjacent unaffected skin (Figure 3C) with 60% and 10% galectin-3 positivity with the intensity of 3+ in cytoplasmic and nuclear patterns.

**Figure 4:**
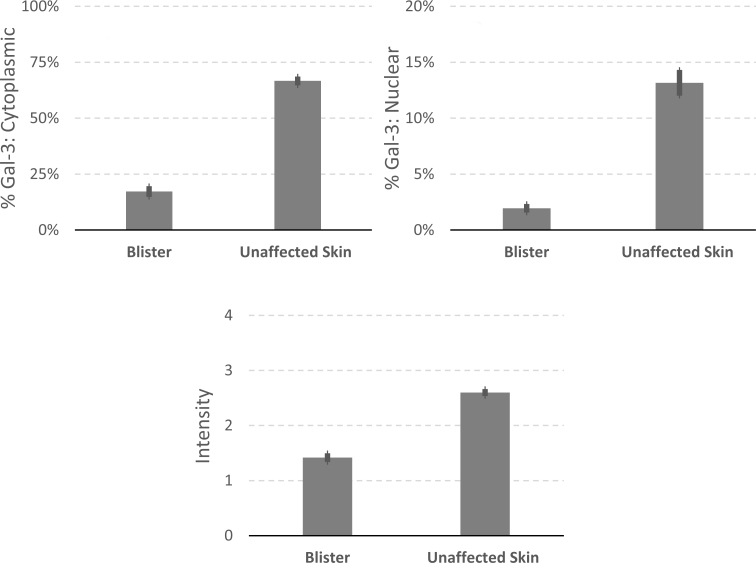
Comparison of galectin-3 expression in blister and adjacent unaffected skin. (A) cytoplasmic expression (P < 0.0001), (B) nuclear expression (P < 0.0001), (C) intensity (P < 0.0001). Gal-3 = galectin-3.

Statistically, there was a significant difference in galectin-3 cytoplasmic expression within keratinocytes immediately surrounding and above the blisters (mean = 17.2% ± 2.4%) compared to those in the adjacent unaffected skin (mean = 66.7% ± 2.0%, P < 0.0001, Figure 4A). Similarly, galectin-3 keratinocyte nuclear expression around blisters (mean = 1.9% ± 0.4%) was significantly lower than in adjacent unaffected skin (mean = 13.2% ± 1.2%, P < 0.0001; Figure 4B). In addition, the cytoplasmic intensity of galectin-3 around blisters (mean = 1.42 ± 0.08) was significantly lower than adjacent unaffected skin (mean = 2.6 ± 0.06, P < 0.0001; Figure 4C).

## Discussion

Galectin-3 is a β-galactoside binding lectin that is found in epithelial cells, including keratinocytes. It is expressed in the nucleus and cytoplasm of normal keratinocytes and related to differentiation and maturation of the keratinocytes [6,7]. Galectin-3 has an important function in cancer development and in inflammatory and immune responses. It has intracellular and extracellular functions, such as playing a crucial role in cell-to-cell interactions. Galectin-3 is expressed in keratinocytes, which is believed to have an impact on the functionality of immune cells in response to inflammatory skin conditions.

The role of galectin-3 in wound healing and various inflammatory skin diseases, such as atopic dermatitis and psoriasis, has been characterized previously. It has been reported that intracellular galectin-3 has an impact on the expression of cell surface and extracellular matrix receptors, which subsequently affects cell-matrix interactions and cell migration. This process has a significant contribution to the healing of wounds [8].

Galectin-3 is a pro-inflammatory mediator in atopic dermatitis, needed for the inflammatory response to epicutaneous antigens via its impact on T cells [9]. Galectin-3 expression in dermal capillaries has a role in the reorganization of the capillary system and the involvement of inflammatory cells in psoriasis [10]. Additionally, it has been shown that galectin-3 has a role in melanocytic and non-melanocytic skin cancers [11]. Galectin-3 promotes cell adhesion, progression and metastasis in melanoma, and its expression is higher in abnormal melanocytes, in contrast to benign, banal melanocytes [12]. Higher expression of galectin-3 in basal cell carcinoma (BCC) and squamous cell carcinoma (SCC) has been observed [11]. It may interact with regulatory genes and influence the development and progression of BCC and SCC. The cytoplasmic expression of galectin-3 is correlated with tumor size in SCC [6].

There has been no report regarding the role of galectin-3 in blister-forming skin disorders such as BP. Urticarial lesions may be one of the first presentations in BP and progress to recurrent chronic blisters. Eosinophils are involved in the pathogenesis of BP and essential for anti-BP2 IgE-mediated blistering. Since galectin-3 is the main element of the IgE group, galectin-3 may have a role in the formation of blisters in this disorder.

Hemidesmosomes are essential structures in keratinocytes for epithelial cell-extracellular matrix connections [13]. Hemidesmosomes are composed of plectin 1a and integrin α6β4, wherein integrin α6β4 functions as a laminin-332 receptor. Galectin-3 stimulates the relationship of laminin-332 and integrin α6β4 [14]. Galectin-3 also promotes epidermal growth factor receptor causing genesis of cell surface proteins. This process is essential to control the cell signal pathway and migration [15].

Blister formation in BP happens as a result of the loss of epithelial cell-extracellular matrix adhesion at the hemidesmosomal junction. Since galectin-3 in keratinocytes regulates cell-extracellular matrix adhesion, its lower expression around blisters in BP may disrupt the hemidesmosomal adhesion complex, resulting in blister formation.

While autoantibodies directed against BP1 and 2 antigens trigger the blister formation, loss of galectin-3 may play a role in the extension of blister formation by initiating cell-extracellular matrix disassembly at the level of the lamina lucida of the basement membrane zone and may also be involved with the inflammatory response and eosinophil chemotaxis underlying the blisters, further accentuating the extent of blister formation.

## Conclusions

We demonstrated lower expression of galectin-3 around the blisters in BP specimens. The pathogenesis of the blister formation may be associated, at least in part, with altered expression of galectin-3. Further studies are required to elucidate the result of this loss on the pathogenesis of the observed histologic findings and determine the exact pathogenesis of blister formation.
